# Influence of nocturnal hypoxemia on follow-up course after type B acute aortic syndrome

**DOI:** 10.1186/s12890-021-01778-y

**Published:** 2021-12-06

**Authors:** Pascal Delsart, Jerome Soquet, Adeline Pierache, Maxime Dedeken, Stephanie Fry, Anne Mallart, François Pontana, Richard Azzaoui, Francis Juthier, Jonathan Sobocinski, Claire Mounier-Vehier

**Affiliations:** 1 Lille, Institut Cœur Poumon, Bd Pr Leclercq, 59000 Lille, France; 2 University of Lille, CHU Lille, 59000 Lille, France; 3 Service d’épidémiologie et de santé publique, University of Lille, CHU Lille, 59000 Lille, France; 4 Vascular Medicine and Hypertension Department, Institut-Coeur-Poumon, Boulevard Pr Leclercq, 59037 Lille Cedex, France

**Keywords:** Aortic dissection, Nocturnal hypoxemia, Sleep apnea, Prognosis

## Abstract

**Introduction:**

Association between sleep nocturnal breathing disorders and acute aortic syndrome (AAS) has been described but mid-term data are scarce.

**Objectives:**

We assessed the prognostic value of sleep apnea parameters and their relationship with aortic morphology after the onset of a type B AAS.

**Methods:**

Between January 2010 and January 2018, sleep apnea screening in post type B AAS was prospectively performed. The association of sleep apnea parameters with aortic morphology and aortic expansion during follow-up was studied.

**Results:**

Over the 8-year-study period, 103 patients were included, with a mean age of 57.8 ± 12.1 years old. Median follow-up was 25.0 months (11.0–51.0). Thirty-two patients (31%) required aortic stenting during the acute phase. In patients treated by aortic stenting, the descending thoracic aortic diameter was positively associated with a higher percentage of nocturnal time of saturation ≤ 90% after adjustment (*p* = 0.016). During follow-up, the nocturnal time of saturation ≤ 90% in patients treated by medical therapy was the only parameter associated with significant aortic expansion rate (r = 0.26, *p* = 0.04). Thirty-eight patients started and sustained nocturnal ventilation during follow-up. The association between aortic expansion rate and nocturnal time of saturation ≤ 90% did not persist during follow-up after adjustment on nocturnal ventilation initiation (r = 0.25, *p* = 0.056).

**Conclusions:**

Nocturnal hypoxemia parameters are positively associated with the max onset aortic diameter and significant aortic growth after type B AAS. Nocturnal ventilation seems to mitigate aortic expansion during follow-up.

**Supplementary Information:**

The online version contains supplementary material available at 10.1186/s12890-021-01778-y.

## Introduction

Prognosis after type B acute aortic dissection remains dreadful nowadays. Despite advances in the management during the acute phase, short and long-term prognoses are still poor [1]. Management during the chronic phase is based on blood pressure control and morphological follow-up of the aorta. Management of hypertension in post type B aortic syndrome is difficult, because most patients suffer from refractory hypertension, associated with different factors of resistance such as obesity, vascular stiffness or sleep apnea syndrome (SAS) [2, 3].

SAS has been described to be highly prevalent in the population with a history of aortic dissection [4, 5]. SAS is associated with aortic wall injury caused by different mechanisms. Nocturnal hypoxemia and nocturnal diastolic blood pressure are associated with an enlargement of the aortic root [4–6]. The apnea–hypopnea index and the oxygen desaturation index are also associated with aortic dilatation at different levels of the aorta [7]. Nevertheless, it is unknown whether SAS only causes progressive aortic dilatation, if it acts as a trigger for aortic dissection onset or if it is responsible for aortic dissection complications.

In this single center study, we prospectively evaluated the association between different parameters of SAS and aortic morphology in post type B acute aortic dissection, with an emphasis on their impact on aortic expansion during follow-up.

## Materials and methods

From January 2010 to January 2018, we prospectively collected the data of patients referred to our institution at the Vascular Medicine and Hypertension unit after type B acute aortic syndrome (the ascending aorta was not involved). The acute nature of the aortic syndrome was defined as a hospital admission before the fourteenth day of the onset of symptoms. Traumatic acute aortic syndromes were not included. Intramural hematomas were considered as aortic dissections with a thrombosed false lumen. After the management of the acute phase, a global cardiovascular assessment was performed 3 to 6 months after discharge. At this time, patients underwent a 24-h blood pressure monitoring and sleep apnea screening.

A 24-h blood pressure monitoring (90207 monitor; Spacelabs Healthcare, Snoqualmie, Washington) was performed with the appropriate cuff size according to the patient’s morphology. The cuff was placed on the dominant arm or on the right arm in the case of thoracic aortic stenting with coverage of the left subclavian artery, and blood pressure was measured every 15 min. Treatment score (number of antihypertensive drugs) was recorded. Our therapeutic strategy followed the recommendations of the European Society of Cardiology, with a blood pressure target below 135/80 mmHg. Clinical and biological cardiovascular risk factors were collected at this time. Connective tissue disease status was defined by the presence of a physician-diagnosed Marfan syndrome (according to the Ghent criteria), documented SMAD 3 mutation or by annulo-aortic ectasia.

All patients underwent screening for SAS by a pneumologist near their place of residence. Daytime sleepiness was evaluated on the Epworth Sleepiness Scale. Obstructive apnea was defined as the cessation of air flow through the nose and mouth for at least 10 s, with the persistence of thoracic or abdominal ventilatory movements during the episode. Central apnea was defined by the cessation of air flow through the nose and mouth for at least 10 s, in the absence of thoracic or abdominal ventilatory movements during the episode. The physicians used the same criteria for hypopnea screening. We used the sleeping time to define the denominator of the event index. The severity of SAS was defined according to the Apnea–Hypopnea Index (AHI) (the number of apnea and hypopnea per hour of sleep) and the Oxygen Desaturation Index (ODI) (the number of times per hour when the blood oxygen level falls by more than 4% compared to the baseline level), the mean nocturnal oxygen saturation, the lowest nocturnal oxygen saturation and the percentage of time per night under 90% of oxygen saturation. A minimum of 4 h of oximetry measure and flow data was required to include a patient in the study.

Computed tomography (CT) angiogram performed before the patients’ discharge from the hospital stay for the acute event was used for the analysis. The diameter of the descending thoracic aorta (at the level of the left superior pulmonary vein) was measured according to Kato et al. [8]. The diameters of the true and false lumens were measured perpendicular to a line running through the 2 insertion points of the intimal flap. The permeability of the false lumen (patent, partially thrombosed or thrombosed) was recorded. The diameter of the ascending aorta was measured by echocardiography at the level of the sinuses of Valsalva. To assess the hypothesis that SAS could be associated with major aortic complications during the acute phase, we chosed to analyze the patients with medical therapy and those with aortic stenting separately. After discharge, a CT angiogram was performed at 3 months, 6 months, 12 months and then annually. The endpoint was the relationship between nocturnal hypoxemia parameters and the aortic expansion rate of the descending thoracic aorta. The aortic dilatation progression rate (mm/year) was defined as (diameterD2-diameterD1)/T, where T was the time (in years) between the CT angiograms recorded for D1 and D2. If an aortic procedure was repeated during follow-up, the CT-angiogram recorded immediately prior to that procedure was taken into consideration in our prognostic analysis. As aortic stenting could affect the aortic expansion during follow-up, we chosed to analyze separately the patients with medical therapy and the patients with aortic stenting.

### Statistical analysis

Continuous variables are expressed as means (standard deviation, SD) in the case of normal distribution or medians [interquartile range] otherwise. Categorical variables are expressed as numbers (percentage). Normality of distributions was assessed using histograms and the Shapiro Wilk test. Due to skewed distribution, some nocturnal hypoxemia parameters were log-transformed for analysis.

We compared the nocturnal hypoxemia parameters between patients treated by aortic stenting and those treated by medical therapy only during the acute phase by using Chi-square tests for binary variables and the Student *t* or the Mann–Whitney U tests (regarding the normality of distributions) for continuous variables. We assessed the associations of sleep apnea parameters with aortic diameters using linear regression models by considering aortic diameters as dependent variables, before and after pre-specified adjustment on age, body mass index, gender, diabetes and the 24-h systolic blood pressure. As exploratory analyses, we performed subgroup analyses according to the acute treatment (medical therapy only vs. aortic stenting). The heterogeneity in the association of sleep apnea parameters with aortic diameters were tested by including the corresponding multiplicative term into regression models. Associations between the aortic dilatation progression rate and nocturnal hypoxemia parameters were analysed using the Spearman’s rank coefficient correlation. Associations were further adjusted for nocturnal ventilation initiation using partial Spearman’s rank coefficient correlation.

We assessed the associations of nocturnal hypoxemia parameters with nocturnal hypertension using the Spearman’s rank or the Pearson coefficient correlation (regarding the normality of distributions). Associations of nocturnal hypoxemia parameters with dipping status was done using analysis of variance.

Statistical testing was conducted at the two-tailed α-level of 0.05. Data were analyzed using the SAS software version 9.4 (SAS Institute, Cary, NC).

## Results

**Fig. 1:**
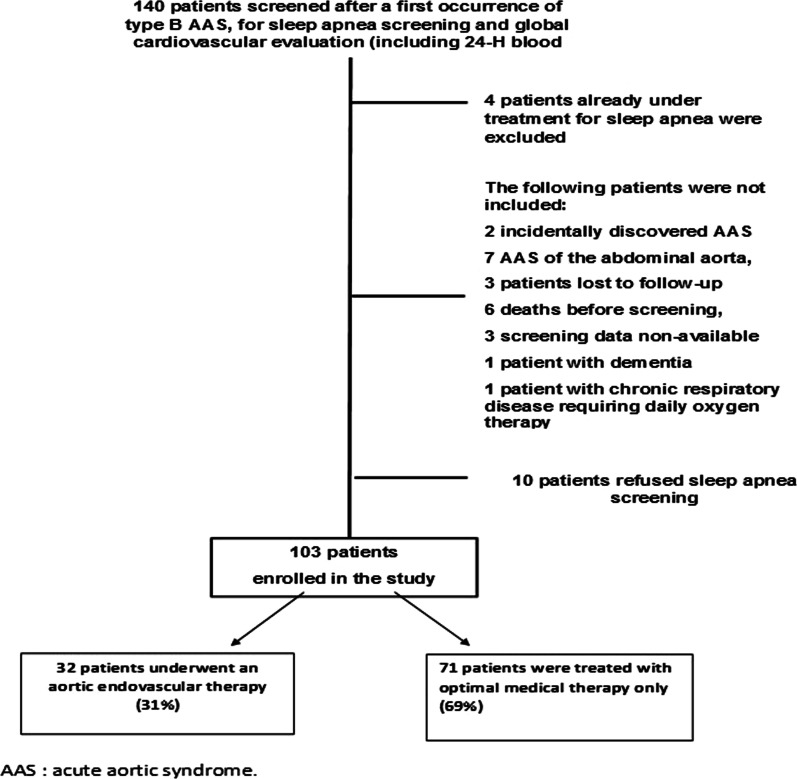
Flow chart of the study population

From January 2010 to January 2018, 103 patients were prospectively included after the onset of a type B acute aortic syndrome. The selection of the study population is summarized in Fig. 1. Four patients were already treated with nocturnal ventilation before the acute phase of the aortic dissection and were excluded from the study. Eight patients were known to have sleep breathing disorder before the occurrence of aortic dissection and were not adherent to the therapy. They benefited from a new sleep apnea screening without treatment and were included in the study. Eighteen patients (17%) had intramural aortic hematoma. Patient characteristics of the study sample are described in Table 1, overall and according to the need of aortic endovascular therapy during the acute phase (aortic stenting in the first month after the onset of the symptoms), which occurred in 32 patients (31%). Twelve patients underwent an aortic intervention because of visceral malperfusion, 15 because of impending rupture and 5 because of refractory pain. There was a trend in the population with medical therapy to have a higher metabolic syndrome profile. Twenty-one (24.7%) patients had chronic obstructive pulmonary disease, with a median force expired volume of 88.5% [77.5–101.1]. Median delay between sleep apnea screening and the onset of the acute aortic dissection was 6 [3–16] months. All devices for sleep apnea screening were type III devices. Eight patients were screened with a Resmed ApneaLink device and the others with polygraphy. Median Epworth scale score was 6 [4–9.5]. Sleep apnea parameters are reported in Table 2, overall and according to the use of aortic stenting. Overall, 94 patients (91.2%) had an AHI greater than 5 per hour, defining SAS. Sixty patients (58%) had an AHI equal or greater than 15 events per hour and 42 patients (41%) had an ODI equal or greater than 15 events per hour. Four patients had central sleep apnea. The mean values of average nocturnal oxygen saturation and of the lowest nocturnal oxygen saturation were of 92.6 ± 2.2% and 82.3 ± 6.5% respectively. The percentage of nocturnal time with a saturation < 90% was 4.0% [0.5 to 18.0%]. As shown in Table 2, compared to patients treated by medical therapy only, patients treated by aortic stenting had higher average nocturnal oxygen saturation values and a lower percentage of nocturnal time with a saturation < 90% (*p* = 0.044). During follow-up, nocturnal ventilation was initiated in 49 patients and 38 of them were adherent to nocturnal treatment. Nocturnal ventilation was started 5.5 [3–18.5] months after the acute aortic dissection onset. In this last subgroup of patients adherent to ventilation, the observance and the efficacy of nocturnal ventilation were checked by collecting data from each patient’s device. The percentage of daily use per week and the number of hours spent per night under treatment were respectively 100% [90–100] and 6.5 [5.3–7.8]. Residual AHI was 0.5 [0.4–3.5].

**Table 1:** Baseline characteristics in overall and according to acute treatment strategy

Characteristics	All patients (n = 103)	Medical therapy only (n = 71)	Aortic stenting (n = 32)	p
*Cardiovascular risk factors*				
Age, years	57.8 (12.1)	59.6 (11.7)	53.8 (12.3)	0.022
Male, n (%)	78 (75.7)	54 (76.1)	24 (75.0)	0.91
BMI, kg/m^2^	28.1 (5.5)	28.8 (5.6)	26.7 (4.8)	0.068
Abdominal circumference^a^, cm	105.5 (14.3)	107.8 (13.3)	100.3 (15.1)	0.014
Current smoking, n (%)	39 (37.9)	27 (38.0)	12 (37.5)	0.96
Diabetes mellitus, n (%)	14 (13.6)	12 (16.9)	2 (6.3)	0.22
Dyslipidemia, n (%)	46 (44.7)	37 (52.1)	9 (28.1)	0.023
Prior cardiovascular disease, n (%)	20 (19.4)	15 (21.2)	5 (15.6)	0.51
Prior aortic surgery, n (%)	19 (18.4)	17 (23.9)	2 (6.3)	0.032
Connective tissue disease, n (%)	15 (14.6)	6 (8.5)	9 (28.1)	0.014
*Biological data*				
Hemoglobin, g/dl, median (IQR)	13.6 [11.7–14.4]	13.5 [11.5–14.4]	13.9 [12.5 –14.4]	0.29
GFR, ml/mn/1.73 m^2^	77.1 (23.4)	75.2 (23.2)	81.4 (23.5)	0.21
LDL cholesterol, g/l, median (IQR)	0.83 [0.67–1.09]	0.80 [0.65–1.00]	0.94 [0.74 –1.15]	0.055
Treatment for hypertension before acute event, n (%)	83 (80.6)	59 (83.1)	24 (75.0)	
*Blood pressure monitoring data, at the time of the scheduled cardiovascular assessment*				
24-h SBP, mmHg	124.1 (14.5)	123.2 (13.7)	126.1 (16.1)	0.35
24-h DBP, mmHg	70.7 (8.0)	71.1 (7.6)	69.9 (8.9)	0.50
Nocturnal SBP, mmHg	120.6 (15.4)	120.4 (15.1)	121.1 (16.3)	0.82
Nocturnal DBP, mmHg	68.0 (8.9)	68.6 (8.9)	66.4 (9.1)	0.25
Non-dipping status, n (%)	83 (80.6)	58 (81.7)	25 (78.1)	0.67
Treatment score, number of hypertensive drugs, median (IQR)	3.0 [3.0–4.0]	4.0 [3.0 –4.0]	[2.0 -4.0]	0.044

BMI, body mass index; GFR, glomerular filtration rate; LDL, low density lipoprotein; SBP, systolic blood pressure; DBP, diastolic blood pressure

^a^5 missing values (1 patient with aortic stent). Values are expressed as mean (standard deviation) unless otherwise as indicated

**Table 2:** Noctural hypoxemia parameters, overall and according to the acute treatment strategy

Ventilatory parameters	All patients (n = 103)	Medical therapy alone (n = 71)	Aortic stenting (n = 32)	*p* value
ODI^a^, number of events per hour, median (IQR)	13.2 [7.0–25.2]	12.6 [6.4–24.9]	14.2 [8.5–27.4]	0.54
ODI^a^, equal or greater than 15, n (%)	42 (45.7)	28 (45.2)	14 (46.7)	0.89
AHI, number of events per hour, median (IQR)	18.2 [9.5–38.2]	18.2 [8.1–36.5]	18.8 [11.0–43.5]	0.66
AHI, equal or greater than 15, n (%)	60 (58.3)	42 (59.2)	18 (56.3)	0.78
Lowest nocturnal oxygen saturation^b^, %	82.3 (6.5)	82.0 (6.7)	82.9 (6.1)	0.56
Mean nocturnal oxygen saturation^c^, %	92.6 (2.2)	92.3 (2.2)	93.2 (1.9)	**0.050**
Percentage of nocturnal time under a saturation of 90%^d^, %, median (IQR)	4.0 [0.5–18.0]	5.9 [1.0–19.0]	1.7 [0.1–12.8]	**0.044**

The results were highlighted (bold) when the *p* value was inferior or equal to 0.05

Values are expressed as mean (standard deviation) unless otherwise indicated

ODI, oxygen desaturation index; AHI, apnea–hypopnea index

^a^11 missing values (2 patients with aortic stenting)

^b^9 missing values (3 patients with aortic stenting)

^c^3 missing values (1 patient with aortic stenting)

^d^4 missing values (1 patient with aortic stenting)

### Nocturnal hypoxemia parameters and aortic diameters

Overall, there were weak correlations between sleep apnea parameters and the ascending aorta diameter in univariate analysis; a positive significant association was found between the ascending aorta diameter and the AHI, as well as the percentage of nocturnal time with a saturation < 90%. However, after pre-specified adjustment for age, body mass index, gender, diabetes and the 24-h systolic blood pressure, these associations were attenuated and did not remain significant (Table 3). After adjustment, only the AHI was associated with the thoracic descending aorta diameter in the whole cohort (*p* = 0.035).

**Table 3:** Associations of nocturnal hypoxemia parameters with aortic diameters in overall study patients

Aortic diameters	Ventilatory parameters	Unadjusted			Adjusted*		
		β (SD)	R^2^	*p* value	β (SD)	R^2†^	*p* value
False lumen TD aorta	ODI^a^	0.209 (1.236)	0.001	0.87	− 0.479 (1.394)	0.039	0.73
	AHI^b^	1.058 (1.002)	0.011	0.29	0.807 (1.079)	0.046	0.45
	Lowest nocturnal oxygen saturation	− 0.233 (0.165)	0.022	0.16	− 0.176 (0.188)	0.047	0.35
	Mean nocturnal oxygen saturation	− 0.521 (0.481)	0.012	0.28	− 0.378 (0.549)	0.045	0.49
	Percentage of nocturnal time under a saturation of 90%^a^	1.069 (0.781)	0.02	0.17	0.928 (0.921)	0.049	0.32
TD diameter of the aorta	ODI^a^	0.601 (1.001)	0.004	0.55	0.941 (1.077)	0.129	0.39
	AHI^b^	1.558 (0.800)	0.037	0.054	1.751 (0.820)	0.159	**0.035**
	Lowest nocturnal oxygen saturation	− 0.033 (0.135)	0.001	0.81	− 0.001 (0.146)	0.123	0.99
	Mean nocturnal oxygen saturation	− 0.224 (0.391)	0.003	0.57	− 0.392 (0.424)	0.133	0.36
	Percentage of nocturnal time under a saturation of 90%^a^	0.593 (0.635)	0.009	0.35	0.682 (0.711)	0.135	0.34
Ascending aorta diameter	ODI^a^	1.102 (0.591)	0.039	0.066	0.559 (0.581)	0.294	0.34
	AHI^b^	1.002 (0.491)	0.042	**0.044**	0.472 (0.449)	0.332	0.3
	Lowest nocturnal oxygen saturation	− 0.052 (0.078)	0.005	0.51	0.042 (0.076)	0.295	0.58
	Mean nocturnal oxygen saturation	− 0.443 (0.230)	0.038	0.057	− 0.133 (0.228)	0.313	0.56
	Percentage of nocturnal time under a saturation of 90%^a^	1.217 (0.361)	0.11	**0.001**	0.570 (0.377)	0.328	0.13

The results were highlighted (bold) when the *p* value was inferior or equal to 0.05

TD, thoracic descending; ODI, oxygen desaturation index; AHI, apnea–hypopnea index

^a^After logarithm + 1 transformation

^b^After logarithm transformation

*Adjusted for age, BMI, sex, diabetes and systolic blood pressure

^†^Partial R squared values calculated in multiple linear regression models

When the association between sleep apnea parameters and aortic diameters were analyzed according to the use of aortic stenting, we found a significant trend heterogeneity in the association between the descending thoracic aorta diameter and most ventilation parameters (Table 4). In patients treated by aortic stenting and after pre-specified adjustment, the descending thoracic aorta diameter was positively associated with the nocturnal percentage of time under of a saturation of 90%. In patients treated by medical therapy only, none of the sleep apnea parameters was associated with the descending thoracic aorta diameter. Although the heterogeneity test across subgroups did not reach the significance level (*p* = 0.061), we found a significant positive association between the AHI and the diameter of the false lumen of the descending thoracic aorta in the stent-treated patients subgroup but not in the medically-treated patients subgroup.

**Table 4:** Associations of nocturnal hypoxemia parameters with aortic diameters according to acute treatment strategy adjusted for age, BMI, sex, diabetes and systolic blood pressure

Aortic diameters	Ventilatory parameters	Population with medical therapy alone, n = 71			Population with aortic stent requirement in acute phase, n = 32			p*
		β (SD)	R^2†^	*p* value	β (SD)	R^2†^	*p* value	
False lumen TD aorta	ODI^a^	− 0.281 (1.496)	0.181	0.85	4.113 (2.607)	0.191	0.13	0.12
	AHI^b^	0.556 (1.089)	0.176	0.61	4.954 (2.142)	0.26	**0.029**	0.061
	Lowest nocturnal oxygen saturation	− 0.236 (0.185)	0.192	0.21	− 0.266 (0.425)	0.119	0.54	0.48
	Mean nocturnal oxygen saturation	− 0.225 (0.550)	0.177	0.68	− 0.547 (1.174)	0.111	0.65	0.81
	Percentage of nocturnal time under a saturation of 90%^a^	0.299 (0.982)	0.176	0.76	1.654 (1.620)	0.14	0.32	0.66
TD diameter of the aorta	ODI^a^	− 0.721 (1.315)	0.136	0.59	3.653 (2.082)	0.323	0.093	**0.018**
	AHI^b^	0.897 (0.942)	0.149	0.34	3.321 (1.851)	0.3	0.085	0.067
	Lowest nocturnal oxygen saturation	0.130 (0.161)	0.156	0.42	− 0.511 (0.327)	0.321	0.13	0.06
	Mean nocturnal oxygen saturation	− 0.227 (0.476)	0.148	0.63	− 1.543 (0.919)	0.296	0.11	0.15
	Percentage of nocturnal time under a saturation of 90%^a^	− 0.227 (0.848)	0.147	0.79	3.111 (1.205)	0.384	**0.016**	**0.016**
Ascending aorta diameter	ODI^a^	1.026 (0.786)	0.145	0.2	0.411 (0.837)	0.673	0.63	0.6
	AHI^b^	0.852 (0.570)	0.201	0.14	− 0.300 (0.729)	0.684	0.68	0.89
	Lowest nocturnal oxygen saturation	0.100 (0.092)	0.154	0.28	− 0.150 (0.127)	0.695	0.25	**0.048**
	Mean nocturnal oxygen saturation	− 0.176 (0.286)	0.164	0.54	0.044 (0.362)	0.675	0.9	0.49
	Percentage of nocturnal time under a saturation of 90%^a^	0.568 (0.503)	0.176	0.26	0.609 (0.492)	0.694	0.23	0.21

The results were highlighted (bold) when the *p* value was inferior or equal to 0.05

Abbreviations. TD: Thoracic descending, ODI: Oxygen desaturation index, AHI: Apnea–hypopnea index

^a^After logarithm + 1 transformation

^b^After logarithm transformation

*For heterogeneity

^†^Partial R squared values calculated in multiple linear regression models

### Nocturnal hypoxemia and aortic expansion rate

After discharge, patients were followed for a median of 25.0 months (11.0 to 51.0) and 39 aortic events occurred during follow-up. Patients who benefited from nocturnal ventilation during follow-up were included in the longitudinal analyses. In the overall population and in the stented population, there was no correlation between nocturnal parameters and the aortic expansion rate during follow-up. In the medical therapy group, the percentage of time < 90% oxygen saturation was the sole parameter associated with the aortic expansion rate (r = 0.26, *p* = 0.04). The lowest nocturnal oxygen saturation tended to be associated with the aortic expansion rate (r = − 0.26, *p* = 0.055). The 3 other parameters (AHI, ODI, mean nocturnal oxygen saturation) were not statistically associated with the aortic expansion rate. After adjustment on nocturnal ventilation initiation, there was no correlation between nocturnal parameters and aortic expansion rate during follow-up overall and in the stented population. The association between aortic expansion rate and nocturnal time of saturation ≤ 90% did not persist after adjustment on nocturnal ventilation initiation during follow-up (r = 0.25, *p* = 0.056).

### Association between nocturnal hypoxemia and nocturnal hypertension

Only a higher percentage of time under a saturation of 90% was significantly associated with higher nocturnal systolic and diastolic blood pressure. The details of the global analysis between sleep breathing disorders and nocturnal hypertension are described in Additional file 1: Table S1.

## Discussion

The novelty of our work is to show an association between nocturnal hypoxemia parameters and the aortic size as well as with the aortic diameter progression in post type B acute aortic syndrome. We have identified two different populations in our study: the population treated by medical therapy only had more severe nocturnal hypoxemia (Table 2) and more severe hypertension, probably due to a more severe overweight phenotype (Table 1); the second group of patients is probably particularly vulnerable to aortic disease. This work is also the first to evoke a potential benefit of nocturnal ventilation to mitigate the harmful effect of nocturnal hypoxemia on aortic expansion during follow-up.

Our study confirms previous reports on the prevalence of severe sleep apnea in the population of patients suffering from aortic dissection [2–5].

The influence of sleep apnea parameters and nocturnal hypoxemia on the descending thoracic aorta morphology has never been studied as far as we are aware of. As Baguet et al.[4], we found an association between nocturnal oxygen saturation parameters and the ascending aorta measures[6]. Hypoxemia is a contributor to atherosclerosis development [9]. Nocturnal hypoxemia may alter the aortic wall and cause aortic enlargement before the acute aortic syndrome. Also, it is likely that patients with a greater aortic size are at higher risk of impending rupture during the acute phase of the aortic syndrome and would benefit the most from an aortic intervention. A history of aortic aneurysm is known to influence the long-term prognosis in post type B acute aortic dissection [10–12]. The association between nocturnal oxygen saturation parameters and the aortic size is strong because it persists even after adjustment on age, body mass index and systolic blood pressure. These latter three parameters are known to progressively increase the aortic size over the years [13]. Obesity and an older age are known to be major risk factors of nocturnal hypoxemia, due to a higher risk of alveolar hypoventilation [14]. In addition, a lower oxygen saturation increases systolic blood pressure [15]. Repetitive desaturations defined by ODI also increase 24-h diastolic blood pressure in post-acute aortic syndrome [16]. The association between a nocturnal percentage of time under a saturation of 90% and the aortic size was only found in the stented group, despite a less severe nocturnal hypoxemia compared to the medical therapy population. We may have identified 2 different phenotypes of patients, as patients in the stented group were more likely to present aortic dissection due to nocturnal hypoxemia harmful effects than patients with medical therapy.

The risk of aortic event remains high during follow-up even in case of aortic intervention during the acute phase [12]. Aortic dissection results from an association between aortic wall injury (entry tear) and systemic factors. Sleep apnea seems to influence the two local mechanisms in relation with aortic disease progression, i.e. false lumen diameter and thrombosis. Sleep apnea seems to be involved in the occurrence of aortic dissection by its influence on the size of the false lumen. In a previous report from a smaller patients sample size, we found an association to explore between sleep apnea parameters and the false lumen diameter in aortic dissections [16]. Likewise, Wand et al. found an association between hypoxemia and partial false lumen thrombosis [17]. Partial false lumen thrombosis has been recognized to influence the prognosis only in post type B acute aortic syndrome [18].

Different mechanisms are described to explain the deleterious association between sleep apnea and aortic dissection: blood pressure surges, increased aortic wall stress and increased sympathetic activity. The mechanical stress due to blood pressure surges following exaggerated negative intra thoracic pressure could explain the severity of type B acute aortic dissectionand the false lumen size. Hypoxemia and apnea are associated with repeat bradycardia resulting from an excessive chemoreflex sensitivity. Bradycardia is followed by an increased stroke volume and thus provokes blood pressure surges. Nocturnal hypoxemia and its different parameters were also associated with aortic size in the acute phase management. But moreover, they showed an influence on aortic expansion during follow-up. The influence of nocturnal hypoxemia on aortic expansion has already been described by Baronne-Rochette et al. in post type A aortic dissection, but the clinical prognosis of sleep apnea parameters in post type B aortic dissection remained unknown so far [2]. In this work, we identified a correlation between the percentage of time under 90% of nocturnal oxygen saturation and the aortic expansion rate. However, unlike Baronne-Rochette et al., we found that nocturnal oxygen saturation was not associated with the progression of aortic dilatation. In our population, intermittent hypoxemia seemed to be more deleterious than a lower mean nocturnal oxygen saturation. Hypoxemia plays directly a role on vascular function impairment. Consequently, nocturnal hypoxemia is thought to cause oxidative stress by decreasing anti-oxidant mechanisms [19].

Increase in oxidative stress following intermittent hypoxemia has been demonstrated in aortic and peripheral artery disease [20]. Oxidative stress effect has been described in the context of abdominal aortic aneurysm. The role of different blood biomarkers of the oxidative are associated with the size of the aneurysm [21, 22]. In the context of abdominal aortic aneurysm, there is also an increase in superoxide anion local production [23]. Therefore, oxidative stress is the result of a local vascular production and systemic factors as smoking in coronary artery disease [24]. It is supposed to have the ability to change the balance between destruction and regeneration of the aortic wall [25]. Therapeutic interventions on oxidative stress in animal models suggest a benefit on the risk of aortic aneurysm expansion and rupture [26]. Intermittent hypoxemia is also linked with endothelial dysfunction, a marker of atherosclerosis. Animal models showed that intermittent hypoxemia is not only a trigger of atherosclerotic lesions, but it also has an independent effect on aortic wall injury. Intermittent hypoxemia leads to an infiltration of aortic wall by macrophages [27] and increases aortic intima-media with elastic fiber alterations and hypertrophy of smooth-muscle cell [28]. Changes in elastin structure in a rat model of intermittent hypoxemia has also been observed [29]. Intermittent hypoxemia provokes an accumulation of collagen fibers and mucoid elements on the aortic wall [30]. Nocturnal hypoxemia also has a harmful effect on systemic factors. It increases blood pressure by sympathetic activation and changes in sympathetic-vagal balance. Hypoxemia is also associated with an increased level of angiotensin II which is a potent vasoconstrictor by directly acting on vascular smooth cells. Nocturnal blood pressure is known to influence long term prognosis in post type B aortic dissection [31].

The present findings are derived from analyses of observational data in a single-center, which are subject to limitations. Sleep apnea assessment were made in different laboratories and the delay between aortic dissection onset and sleep apnea testing is relatively long. We also caution that we cannot exclude a lack of adequate statistical power to detect a difference, considering our small study sample size. Finally, the influence of blood pressure during follow-up was not evaluated in the present study.

Nocturnal hypoxemia may influence the acute onset of type B aortic dissection and the aortic expansion rate. Nocturnal hypoxemia per se seems to impact the prognosis during follow-up, in patients who were only treated medically during the acute phase. Initiation of a nocturnal ventilation seems to mitigate aortic expansion during follow-up in a population of patients highly adherent to their nocturnal treatment. These findings should be integrated in further studies evaluating the prognosis of patients who are not stented after a type B aortic dissection. Endovascular technics should protect patients from adverse effects of nocturnal hypoxemia.

## Supplementary Information

**Additional file 1: Table S1.** Associations between ventilatory parameters and nocturnal hypertension, dipping status.

## Data Availability

The datasets used and/or analyzed during the current study are available from the corresponding author on reasonable request.
