# Supplementary material for: Influence of nocturnal hypoxemia on follow-up course after type B acute aortic syndrome
Source: BMC Pulm Med. 2021 Dec 6;21:401. doi: 10.1186/s12890-021-01778-y (PMC8647351; doi:10.1186/s12890-021-01778-y)
Supplement: Supplementary file 1 — Additional file 1: Table S1. Associations between ventilatory parameters and nocturnal hypertension, dipping status. [file 12890_2021_1778_MOESM1_ESM.docx]

|  | **ODI** | | **AHI** | | **Lowest nocturnal oxygen saturation** | | **Mean nocturnal oxygen saturation** | | **Percentage of nocturnal time under a saturation of 90%** | |
| --- | --- | --- | --- | --- | --- | --- | --- | --- | --- | --- |
| **Continuous Factors** | **Spearman Correlation** | **P-value** | **Spearman Correlation** | **P-value** | **Pearson Correlation** | **P-value** | **Pearson Correlation** | **P-value** | **Spearman Correlation** | **P-value** |
| Nocturnal-SBP  Nocturnal-DBP | 0.05  0.11 | 0.65  0.29 | 0.19  0.12 | 0.061  0.23 | -0.06  -0.11 | 0.59  0.27 | -0.09  -0.04 | 0.38  0.67 | 0.20  0.21 | **0.047**  **0.039** |
| **Categorical Factors** | **Median [IQR]** | **P-value^1^** | **Median [IQR]** | **P-value^2^** | **Mean (SD)** | **P-value** | **Mean (SD)** | **P-value** | **Median [IQR]** | **P-value^1^** |
| Dipping status No  Yes | 13.3 [5.6 -25.5]  13 [10.3 -24] | 0.26 | 18 [8 -43]  18.6 [12.6 -36.3] | 0.39 | 82.4 (6.7)  81.7 (5.8) | 0.67 | 92.7 (2.1)  92.1 (2.3) | 0.22 | 3.4 [0.5 -11.7]  16.4 [1.4 -33.9] | 0.073 |

**Supplementary Table 1.** Associations between ventilatory parameters and nocturnal hypertension, dipping status.

Abbreviations. AHI : Apnea-hypopnea index

^1^ : after logarithm+1 transformation, ^2^ : after logarithm transformation.

We assessed the associations of ventilatory parameters with nocturnal hypertension using the Spearman’s rank or the Pearson coefficient correlation (regarding the normality of distributions). Associations of ventilatory parameters with dipping status was done using analysis of variance.
